# Direct Laser Interference Patterning of Diffraction Gratings in Safrofilcon-A Hydrogel: Fabrication and Hydration Assessment

**DOI:** 10.3390/polym13050679

**Published:** 2021-02-24

**Authors:** Daniel Sola, Stephan Milles, Andrés F. Lasagni

**Affiliations:** 1 Institut für Fertigungstechnik, Technische Universität Dresden, 01069 Dresden, Germany; stephan.milles@tu-dresden.de (S.M.); andres_fabian.lasagni@tu-dresden.de (A.F.L.); 2 Laboratorio de Óptica, Centro de Investigación en Óptica y Nanofísica, Campus Espinardo, Universidad de Murcia, 30100 Murcia, Spain; 3 Fraunhofer Institut für Werkstoff- und Strahltechnik IWS, Winterbergstr. 28, 01277 Dresden, Germany

**Keywords:** DLIP, laser materials processing, diffraction gratings, ophthalmic materials, polymers

## Abstract

Refractive index modification by laser micro-structuration of diffractive optical devices in ophthalmic polymers has recently been applied for refractive correction in the fields of optics and ophthalmology. In this work, Safrofilcon-A hydrogel, used as soft contact lenses, was processed by direct laser interference patterning (DLIP) to fabricate linear periodic patterns on the surface of the samples. Periodic modulation of the surface was attained under two-beam interference by using a Q-switched laser source with emission at 263 nm and 4 ns pulse duration. Features of processed areas were studied as a function of both the interference spatial period and the laser fluence. Optical confocal microscopy used to evaluate the topography of the processed samples showed that both structured height and surface roughness increased with laser fluence. Static water contact angle (WCA) measurements were carried out with deionized water droplets on the structured areas to evaluate the hydration properties of DLIP structures. It was observed that the laser structured areas induced a delay in the hydration process. Finally, microstructural changes induced in the structured areas were assessed by confocal micro-Raman spectroscopy showing that at low laser fluences the polymer structure remained almost unaltered. In addition, Raman spectra of hydrated samples recovered the original shape of areas structured at low laser fluence.

## 1. Introduction

Polymer technology has rapidly advanced since the beginning of this century. This development has provided polymers with excellent properties, such as high optical transparency in the UV-visible-NIR spectral region, elasticity, flexibility, durability [1,2,3,4], oxygen permeability, hydrophobicity, biostability, and biocompatibility [5,6,7,8]. In addition, manufacturing process is easy, reliable, and highly efficient. Polymers are currently the preferred materials in almost all biotechnological applications. In particular, in biomedicine, they have been applied in cardiovascular devices [6], drug delivery [7], as hard and soft tissue replacement [8], and as both contact and intraocular lenses [8,9,10]. 

Short and ultrashort pulsed laser radiation has been recently used to structure polymers, crystals, and glasses in applications such as 2D/3D micro/nanostructures [11,12], active and passive waveguides [13,14,15,16,17,18], photonic crystals [19,20], beam splitters [11,19], data storage elements [11,19,21], and microfluidic components [19,22,23]. In addition, in ophthalmology, ultrashort direct laser writing (UDLW) has been applied to vision correction in photo-refractive surgery [24,25,26], and more recently, to change the power of refractive optical elements [27,28,29,30,31,32,33,34]. It is well known that diffractive optical elements, such as diffraction gratings, can be used to modify the refractive index and hence the refractive power of an optical device. Specifically, ultrashort laser radiation with laser pulse energy below damage threshold has been employed to fabricate diffraction gratings within dye-doped and non-doped ophthalmic polymers, resulting in refractive index modification ranging from ~6 × 10^−2^ to ~8 × 10^−2^ [27,28,29,30,31,32,33,34]. Nonetheless, the processing rates reported to date to structure areas of large dimensions, such as the cornea, are low, which hinders their application on a real scale. This limitation can be overcome if the whole pattern, instead of line by line, is transferred to the sample at once. This can be achieved using direct laser interference patterning (DLIP). We have recently proposed DLIP as a novel approach to fabricate diffraction gratings on the surface of ophthalmic polymers to be used for refractive correction [35,36,37]. DLIP is a single-step and non-contact laser processing technique, which is more flexible and cost-effective when compared to traditional structuring techniques in the micro- and sub-micrometer range [37,38,39,40,41,42]. To date, we have achieved refractive index changes one order of magnitude higher, with processing yields more than two orders of magnitude faster, than those reported so far by UDLW in similar non-doped ophthalmic polymers [35,36,37]. 

Ophthalmic polymers for soft contact lenses are commonly machined in dry stage by lathe cutting to provide them with the required refractive power. Next, they are hydrated by immersion in saline solution for 24 h, providing them the suitable flexibility to be placed over the corneal epithelium. To date, our investigations on DLIP structuring in ophthalmic polymers have been carried out in the dry stage. Nevertheless, accounting for the fact that the final sample is softened by hydration, it is important to assess to what extent the laser structuring modifies the capability of the hydrogel to be hydrated. This evaluation will provide the limiting laser processing parameters to modify the refractive index. 

In this work, we investigate how wettability and consequently the hydration process of the polymer sample may be affected by the laser-induced surface patterning. For this purpose, periodic patterns are fabricated by means of DLIP under two-beam configuration with a pulsed laser emitting at 263 nm, and pulsewidth in the nanosecond range. Confocal microscopy and micro-Raman spectroscopy are performed to investigate surface topography, and compositional and structural changes in the laser-processed areas. Finally, static water contact angle (WCA) measurements are carried out with deionized water droplets on the structured areas to evaluate the hydration properties of DLIP structures.

## 2. Experimental System

### 2.1. Laser Setup

**Figure 1:**
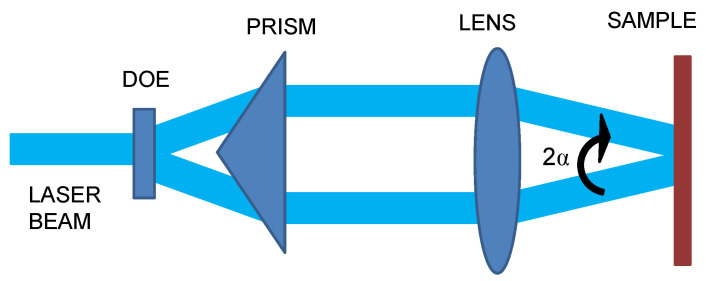
Schematic representation of direct laser interference patterning (DLIP) setup.

As the laser source, a Q-Switched Nd:YAG laser emitting at 263 nm with pulsewidth of 4 ns and repetition rate of 1 kHz was used to fabricate the periodic structures (Laser-export Co. Ltd., Tech-263 Advanced, Moscow, Russia). Laser beam was split into two beams of equal intensity by using a diffractive optical element (DOE), and both laser beams were collimated by means of a prism. An optical lens of 60 mm focal length was used to interfere both laser beams on the surface of the sample, as shown in Figure 1. Angle between the laser beams, 2*α*, and the wavelength of the laser radiation, *λ*, allowed one to control the interference period, Λ, according to the following equation [38]:

(1) $$\Lambda =\frac{\lambda }{2sin\alpha }\;,$$

Interference spatial periods were experimentally set at 3 µm and 6 µm. Laser fluence was set at 0.47 J/cm^2^, and number of pulses was modified between 2 and 10 pulses. These values were selected after previous experiments to be above the modification threshold and not to induce an excessive damage on the samples.

### 2.2. Materials

**Figure 2:**
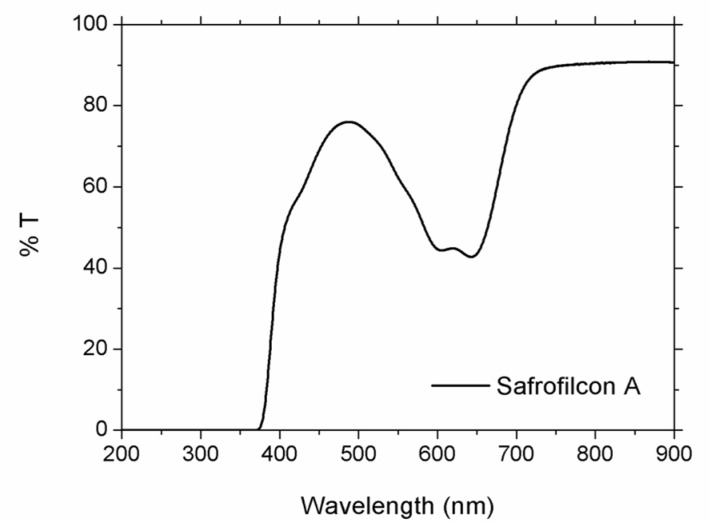
Optical transmission spectra of Safrofilcon-A hydrogel polymer.

As the substrate, 1 mm thick Safrofilcon-A hydrogel polymer disks, provided by the manufacturer (Contamac Ltd., Saffron Walden, UK) in dry stage, were used to be processed. The optical transmission spectrum is shown in Figure 2. 

### 2.3. Characterization Techniques

Optical transmission spectra were obtained by means of a spectrophotometer (U-3400, Hitachi, Abingdon, UK). Optical confocal microscopy (Sensofar S Neox, Terrassa, Spain) was performed to investigate surface topographies and height profiles of the structured samples by using a 150× microscope objective, which provided an optical resolution of 140 nm and a lateral resolution of 1 nm. Surface roughness was determined by using the arithmetical mean height of the surface, Sa. Confocal micro-Raman spectroscopy was utilized to characterize the microstructural changes using a confocal optical microscope coupled to a spectrometer (SR303i-B, Andor, Belfast, Northern Ireland), equipped with a thermoelectric-cooled CCD detector (Newton 920, Andor, Belfast, Northern Ireland). A continuous wave 785 nm laser was used as the excitation source. Laser power was kept below 50 mW to avoid the heating of the sample. The backscattered light was collected through a 60× (0.85 NA) microscope objective lens. Finally, static water contact angle (WCA) measurements were performed using a drop shape analyzer (Krüss DSA 100 S, Hamburg, Germany) and a tensile droplet volume of 2 µL at ambient conditions of 22 °C and 16% of relative humidity. The tangent droplet fitting method was used for all measurements to determine the contact angles between the surface and the droplet. Each measurement was repeated three times for a statistical purpose.

## 3. Results and Discussion

### 3.1. DLIP Structuring

**Figure 3:**
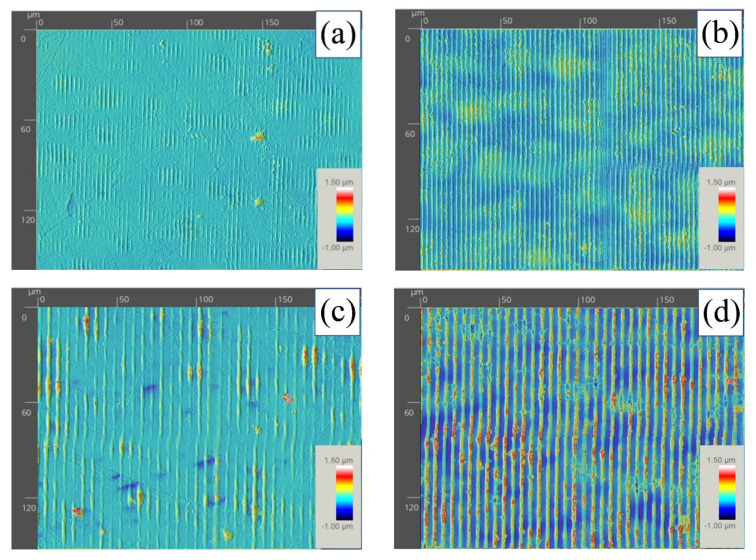
Confocal topographies of structured samples with spatial periods Λ of 3 µm (**a**,**b**) and 6 µm (**c**,**d**) using 2 (left) and 6 (right) laser pulses of 0.47 J/cm^2^.

The polymer samples were structured with periodic line-like patterns adjusting the experimental setup according to equation 1 to induce spatial periods of 3 µm and 6 µm on the surface of the sample. Multi-pulse laser structuring was carried out delivering 2, 4, 6, 8, and 10 laser pulses at a laser fluence of 0.47 J/cm^2^. Previous experiments allowed one to determine this laser fluence as optimal to process this material. Lower and higher laser fluences were found to be unsuitable since neither produced any effect on the material nor induced significant damage. Figure 3 shows surface topographies of structured samples with spatial periods Λ of 3 µm (a and b) and 6 µm (c and d) using 2 (left) and 6 (right) laser pulses of 0.47 J/cm^2^. Experimental period of the interference pattern was assessed by confocal microscopy, resulting in 3.03 ± 0.22 µm and 6.10 ± 0.16 µm, close to the theoretical values given by Equation (1). It can be observed that the higher the number of pulses, the more material was re-deposited on the surface of the material, thus leading to a more undefined structure. Accounting for the fact that the pulse duration of this laser source was in the nanosecond range, and the high optical absorption of this polymer at the laser wavelength was used to carry out the process (263 nm), the laser intensity was transferred onto the material by both photo-chemical and photo-thermal processes. This type of laser-matter interaction implies direct bond breaking and thermally induced vaporization processes [43].

**Figure 4:**
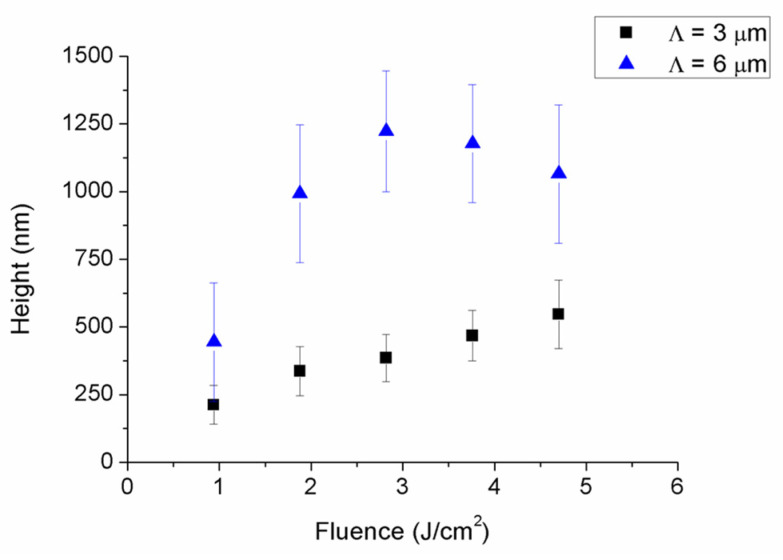
Height of DLIP structures as a function of delivered cumulated laser fluence.

**Figure 5:**
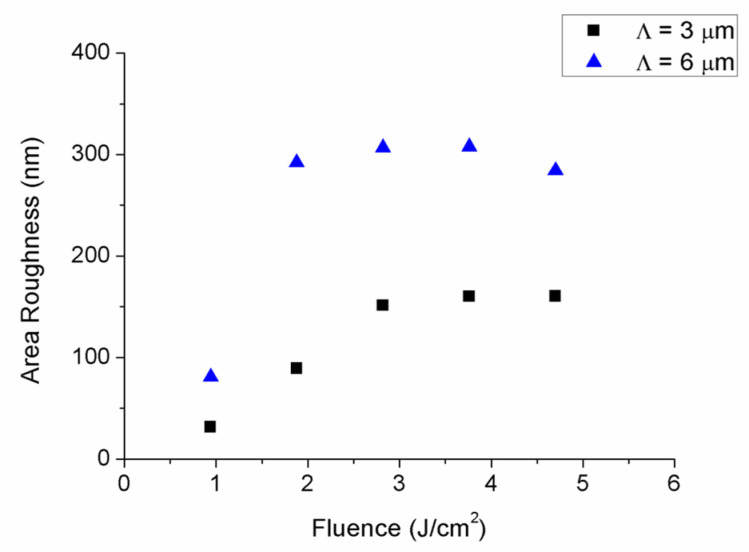
Surface roughness of structured samples as a function of delivered fluence.

Next, profile measurements were taken by using confocal microscopy to evaluate the height of DLIP structures. As Figure 4 shows, structured height increased with laser fluence. For the case of samples structured with a spatial period of 3 µm, the increase was found to be linear. Nevertheless, in samples structured with a spatial period of 6 µm, the maximal height was achieved at a cumulated laser fluence of 2.82 J/cm^2^ (6 laser pulses of 0.47 J/cm^2^). Higher laser fluences led to a lower height due to both an increased damage induced by the laser radiation in the polymer and to the greater amount of material re-deposited onto the surface as a consequence of the laser ablation process. It is worth highlighting that the height of DLIP structures decreased with the spatial period, in good agreement with previous works found in the literature [41]. In addition to the DLIP height, surface roughness (Sa) was assessed by confocal microscopy, shown in Figure 5. It was observed that roughness was relatively high compared to the structure depth, and that increased with laser fluence until reaching a saturation value. This value was around 160 nm and was achieved with six laser pulses for samples structured with a spatial period of 3 µm, whereas this saturation value was higher and was reached earlier for samples processed with a spatial period of 6 µm. Specifically, it was found to be around 300 nm and was achieved with 4 laser pulses. 

### 3.2. Hydration Assessment

**Figure 6:**
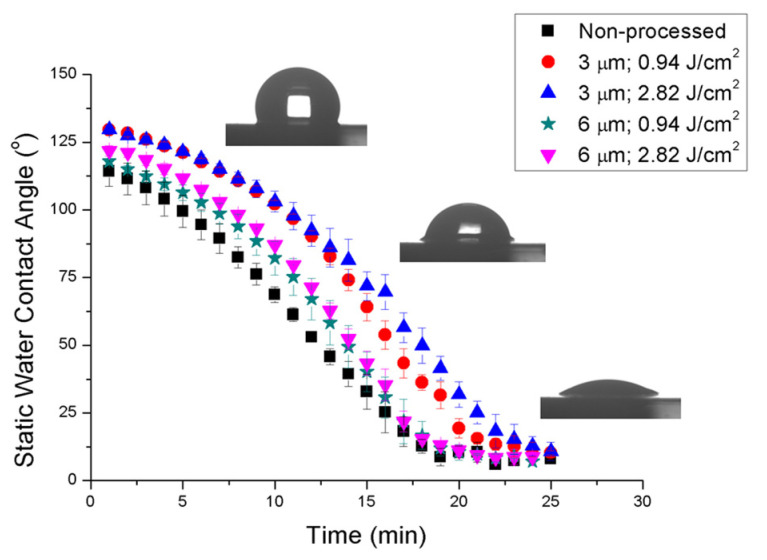
Time-dependent static water contact angle measurements performed during the absorption of the water droplet by the polymer samples.

Static water contact angle (WCA) measurements were performed to evaluate the wetting characteristics of the laser-structured areas. Figure 6 shows time-dependent measurements taken during the absorption of the water droplet by the polymer samples. It can be observed that all samples showed the same behavior; departing from the initial WCA value, it decreased as a function of time until an inflection point appeared for a WCA value around 9°. Once this point was reached, the curve became flat. For non-processed samples to reach this inflection point took around 19 min. Concerning DLIP-structured samples, the most significant parameter affecting the hydration process was the spatial period. Provided a spatial period, to reach the inflection point took approximately the same time independently of the laser fluence used to structure the sample, specifically, around 22 min and 24 min for samples structured with spatial periods of 6 µm and 3 µm, respectively. Therefore, it was found that hydration process was modified by the laser-induced DLIP structures so that the smaller the spatial period, the longer the time for the sample to be hydrated. In particular, the delay time was estimated in 15% and 26% for Λ of 6 µm and 3 µm, respectively. In addition, although both non-processed and DLIP-structured samples were shown to be hydrophobic, it was observed that the value of the WCA was also affected by the structuring period. For instance, regarding the initial value of the WCA, in non-processed samples it took values around 114° whereas for DLIP-structured samples it took values around 121° and 129° for Λ of 6 µm and 3 µm, respectively. It was also observed that WCA value was also affected by the laser fluence at which the laser structuring was carried out, so that it was increased with the laser fluence. This increase could reach up to a 7% and a 30% in samples structured with spatial periods of 6 µm and 3 µm, respectively, when increasing the cumulated laser fluence from 0.94 J/cm^2^ to 2.82 J/cm^2^.

### 3.3. Microstructural Characterization

**Figure 7:**
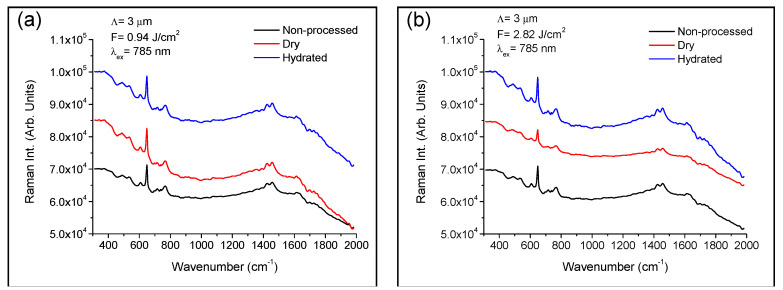
Micro-Raman spectra of the polymer sample in non-processed areas and in the DLIP structured regions with a spatial period of 3 µm at 0.94 J/cm^2^ laser fluence (**a**) and 2.82 J/cm^2^ laser fluence (**b**) before and after hydration assessment.

Confocal micro-Raman spectroscopy was performed in laser-structured areas as in dry stage after hydration assessment to investigate modifications in both polymer structure and chemical composition. Figure 7 shows Raman spectra in the wavenumber region 300–2000 cm^−1^ of the polymer sample in non-structured regions and in the DLIP processed areas with a spatial period of 3 µm at 0.94 J/cm^2^ (a) and 2.82 J/cm^2^ of cumulated laser fluence (b) before and after hydration assessment. Raman spectra showed sharp peaks and broad bands, which agreed with those previously reported in the literature [44,45]. These peaks and bands were assigned as follows: 605 cm^−1^, νsCCO; 646 cm^−1^, SiO_3_; 766 cm^−1^, SiCH_3_; 1425 cm^−1^ δCH_2_; 1457 cm^−1^ δCH_2_ and δCH_3_; and 1615 cm^−1^ νCO. It is observed that Raman spectra of areas structured at low cumulated laser fluence, 0.94 J/cm^2^, before and after hydration did not show significant changes when compared with non-processed areas, Figure 7a. Therefore, at low laser fluences the polymer structure remained almost unaltered and hydration process did not produce any substantial modification. However, areas structured at higher fluences showed a strong decrease in the intensity of Raman peaks placed at 646 cm^−1^, 766 cm^−1^, 1425 cm^−1^, 1457 cm^−1^, and 1615 cm^−1^, as shown in Figure 7b. This modification resulted from the photo-thermal damaged induced by the laser radiation. It is worth noting that Raman spectra of these samples after hydration process recovered the original shape of areas structured at low laser fluence, as shown in Figure 7b. 

## 4. Conclusions

Safrofilcon-A hydrogel polymers employed as soft contact lenses were structured on the surface with linear periodic patterns using DLIP with UV pulsed laser radiation in the nanosecond range. The produced periodic patterns were evaluated as a function of the cumulated laser fluence and the spatial period. It was found that height of the DLIP patterns increased with the cumulated laser fluence (or pulse number at a constant fluence). In addition, the height of the periodic structure decreased when the spatial period decreased. Additionally, it was observed that surface roughness increased with both laser fluence and spatial period. Evolution of static water contact angle (WCA) as a function of time was assessed to study how DLIP structures may affect the hydration of the polymer sample. Static water contact angle measurements showed that WCA decreased as a function of time and became flat for a contact angle around 9°. In addition, the laser structured samples induced a delay in the hydration process, so that the shorter the spatial period, the longer the time required for the sample to be hydrated. Delay time was estimated in 15% and 26% for Λ of 6 µm and 3 µm, respectively. Contact angle value was affected by the structuring period. It increased for structured samples, so that the larger the spatial period, the lower the contact angle. Micro-Raman analyses carried out in the processed areas showed that at low cumulated laser fluence polymer structure remained almost unaltered. However, high laser fluence induced photo-thermal damaged on the polymer sample. Furthermore, Raman analyses performed after hydration process showed that structured samples recovered the spectra of areas structured at low laser fluence.

## Data Availability

The data presented in this study are available on request from the corresponding author.
